# Depletion of essential fatty acids in muscle is associated with shorter survival of cancer patients undergoing surgery-preliminary report

**DOI:** 10.1038/s41598-021-02269-0

**Published:** 2021-11-26

**Authors:** Amritpal S. Bhullar, Irma Magaly Rivas-Serna, Ana Anoveros-Barrera, Abha Dunichand-Hoedl, David Bigam, Rachel G. Khadaroo, Todd McMullen, Oliver Bathe, Charles T. Putman, Vickie Baracos, Michael T. Clandinin, Vera C. Mazurak

**Affiliations:** 1 Department of Agricultural, Food & Nutritional Science, University of Alberta, 4-002 Li Ka Shing Centre for Health Research Innovation, 8602-112 St NW, Edmonton, AB Canada; 2 Department of Surgery, University of Alberta, Edmonton, Canada; 3 Departments of Surgery and Oncology, Tom Baker Cancer Centre, University of Calgary, Calgary, Canada; 4 Faculty of Kinesiology, Sport, and Recreation, University of Alberta, Edmonton, Canada; 5 Department of Oncology, University of Alberta, Edmonton, Canada; 6 Department of Medicine, University of Alberta, Edmonton, Canada

**Keywords:** Lipids, Cancer, Gastrointestinal cancer

## Abstract

Emerging studies are reporting associations between skeletal muscle abnormalities and survival in cancer patients. Cancer prognosis is associated with depletion of essential fatty acids in erythrocytes and plasma in humans. However the relationship between skeletal muscle membrane fatty acid composition and survival is unknown. This study investigates the relationship between fatty acid content of phospholipids in skeletal muscle and survival in cancer patients. *Rectus abdominis* biopsies were collected during cancer surgery from 35 patients diagnosed with cancer. Thin-layer and gas chromatography were used for quantification of phospholipid fatty acids. Cutpoints for survival were defined using optimal stratification. Median survival was between 450 and 500 days when patients had arachidonic acid (AA) eicosapentaenoic acid (EPA) and docosahexaenoic acid (DHA) in muscle phospholipid below the cut-point compared to 720–800 days for patients above. Cox regression analysis revealed that low amounts of AA, EPA and DHA are risk factors for death. The risk of death remained significant for AA [HR 3.5 (1.11–10.87), p = 0.03], EPA [HR 3.92 (1.1–14.0), p = 0.04] and DHA [HR 4.08 (1.1–14.6), p = 0.03] when adjusted for sex. Lower amounts of essential fatty acids in skeletal muscle membrane is a predictor of survival in cancer patients. These results warrant investigation to restore bioactive fatty acids in people with cancer.

## Introduction

Essential fatty acids incorporated into membranes influence cancer progression by altering production of lipid mediators, influencing gene expression as well as activating signal transduction molecules influencing carcinogenesis^1,2^. Experimental studies of cancer show that arachidonic acid (AA, 20:4n-6), eicosapentaenoic acid (EPA, 20:5n-3) and docosahexaenoic acid (DHA, 22:6n-3) regulate proliferation, apoptosis, cytotoxicity, metastasis and immune cell functions in cancer cell lines^3^. EPA and DHA improve efficacy of anticancer treatments, chemotherapy tolerability, and inflammatory profile of the host while attenuating skeletal muscle mass depletion^4,5^, although the quality of studies is limited^6^. Collectively, the metabolic properties of essential fatty acids acting on tumor and host may influence survival of cancer patients.

Alterations in phospholipid fatty acid composition of adipose tissue, erythrocytes, neutrophils and blood plasma occur in patients with lung, colorectal and pancreatic cancers^7–12^. Specifically, a decrease in AA, EPA and DHA has been reported in the blood phospholipids of cancer patients compared to healthy individuals, independent of changes in caloric and fat intake^7,13–15^. Higher content of palmitoleic (16:1) and oleic acid (18:1) concurrent with a lower content of AA and linoleic acid (18:2n-6) has been observed in erythrocytes of patients with lung adenocarcinoma and small cell lung cancer compared to healthy controls^7^. Shorter survival of lung, pancreatic and colorectal cancer patients exhibiting lower levels of essential fatty acids in blood plasma has been observed^9,16^. Murphy et al. reported 59% lower AA, 26% lower EPA, and 40% lower DHA in plasma phospholipids of cancer patients who survived fewer than the median survival of 238 days compared to patients who survived > 238 days^9^. Having a greater proportion of EPA and DHA in plasma phospholipids associates with better prognosis (survival more than 100 days) in pancreatic cancer patients compared to patients with lower proportions^16^. A recent study reported lower AA in serum phospholipids of colorectal patients who exhibited disease progression after one year follow up compared to individuals without tumor progression^13^. Overall, measures in plasma phospholipids suggest that depletion of essential fatty acids is associated with shorter survival in cancer patients.

EPA and DHA supplementation attenuate depletion of skeletal muscle mass in individuals with cancer, a well-established prognostic factor^17–20^. EPA and DHA are lower in plasma phospholipids of patients with sarcopenia compared to patients with normal muscle mass^9^. The highest concentrations of EPA and DHA in plasma phospholipids were observed in lung cancer patients who maintained or gained skeletal muscle during cancer treatment^9^. Several studies have shown an association between attenuation of muscle loss and n-3 fatty acid supplementation, however, these studies assessed fatty acid composition of plasma only and not of the muscle membrane^17,19^.

No investigation into the alteration in fatty acid composition of phospholipids of skeletal muscle in cancer patients has been conducted. Based on observations in blood phospholipids, reduced levels of AA, EPA and DHA in skeletal muscle phospholipids are likely to be associated with shorter survival of cancer patients. The objective of the study was to investigate the relationship between fatty acid content of phospholipids in skeletal muscle and survival in patients with gastrointestinal cancers who provided biopsy during surgery. It was hypothesized that patients with higher levels of essential fatty acids in muscle phospholipids would have a better prognosis.

## Materials and methods

### Ethics statement

The study was approved by the Health Research Ethics Board of Alberta-Cancer and abides by the Declaration of Helsinki principles. Patients undergoing elective abdominal surgery were consecutively approached between Jun 2015 and Sept 2017 to participate in tumor and tissue banking at a hepatopancreatobiliary surgical service in Alberta, Canada. Ninety three percent of patients approached agreed to participate. Patients provided written informed consent for muscle biopsy and tissue banking and patient information (demographic, clinical, and surgical data) from medical records. The release of 35 samples from the tissue bank for analysis, as well as patient information (demographic, clinical and operative data) from medical records, was performed under the auspices of Protocol ETH-21709: *The Molecular Profile of Cancer Cachexia*.

### Subjects and muscle biopsies

*Rectus abdominis* biopsies of 35 patients undergoing surgery for colorectal, pancreatic and other gastrointestinal tumors were studied. The study cohort and conditions for acquisition of muscle samples have been described previously^21^. Patient characteristics are shown in Table 1.

**Table 1:** Patient characteristics.

Characteristic	All patients
N	35
Age (years)	64.4 ± 10.8
BMI (kg/m^2^)	27.1 ± 7.1
Male:Female	24:11
**Cancer types, N (%)**	
Colorectal	14 (40)
Pancreatic	9 (26)
Liver	5 (14)
Other Gastro-intestinal^a^	7 (20)
Metastasis, N (%)	21 (63)
Median Overall Survival (days)	616
**CT-image measures at L3**^**b**^	
Skeletal Muscle Index (cm^2^/m^2^)	45.5 ± 9.2
Muscle Radiodensity (HU)	30.1 ± 9.2
**Comorbidities, N (%)**	
Diabetes Type II	10 (28)
Cardiovascular Disease	12 (34)
Dyslipidemia	9 (26)
Smoking habit, N (%)	14 (40)

Values are mean ± SD.

^a^Other GI cancer includes gastric and gall bladder cancer.

^b^CT measurements available for 33 patients.

### CT image analysis

CT scans completed with a spiral CT scanner for initial cancer staging and routine diagnostic purposes were used to quantify muscle area, cross section area, adipose tissue area and mean skeletal muscle radiodensity to confirm that the population sampled is representative of mean values for cancer population^22^. CT scans completed before surgery were analysed using SliceOmatic V4.2 software with CT image parameters that include: contrast, 5 mm slice thickness, 120 kVP, and 290 mA. Total skeletal muscle area (cm^2^) was evaluated on a single image at the third lumbar vertebrae (L3) using Hounsfield unit (HU) thresholds of − 29 to 150 for skeletal muscle. Total skeletal muscle area was normalized for stature (m^2^) and reported as skeletal muscle index (SMI) (cm^2^ m^2^). Mean muscle radiodensity (HU) is reported for the entire muscle area (i.e., quadratus lumborum, psoas, erector spinae, external obliques, transverse abdominis, internal obliques, and rectus abdominis).

### Analysis of phospholipids by gas chromatography

The biopsy [≈ 50 mg] was ground using a frozen pestle and mortar without letting the muscle tissue thaw. Ground tissue was homogenized in calcium chloride [CaCl_2_; 0.025%] solution. A modified Folch method was used to extract lipids from muscle^9,12^. Lipids were extracted using chloroform/methanol (2:1, vol/vol). Thin layer chromatography chromatography (TLC) plates (G plated, Silica Gel, 20 × 20 cm, 250 microns, Analtech Inc., Newark, DE) was used to isolate phospholipids (PL). Solvent (80:20:1 petroleum ether/diethyl/ethyl ether/acetic acid [glacial; HAC]) was added to the developmental chamber. Chloroform/methanol (2:1) was added to each dried tube, vortexed and samples were spotted on plates in duplicate. Spotted plates were run in a solvent system until the solvent mixture reached ~ 1.5 cm from the top of the plate. Dried plates were sprayed with 0.1% 8-anilino-1-naphthalenesulfonic acid (ANSA) to visualize the PL bands under ultraviolet light. Bands were scraped andan internal standard, C17:0, was added for PL fatty acid quantification followed by methylation. PL fatty acid composition was analysed by gas chromatography-flame ionisation detector in a Varian 3900 gas chromatography [Varian Instruments, Georgetown, ON, Canada]. The quantity of fatty acids within the PL fraction was calculated by comparison with the known concentration of the internal standard and the sum of all fatty acids was reported as total PL. The peak area of each fatty acid was normalized against the sum of peak areas of all fatty acids to determine the relative proportions of fatty acids comprising the PL fraction.

### Statistical analysis

The primary outcome was overall survival, defined as the number of days surviving after the initial visit by each patient. Patients were observed until their deaths or until November 30, 2018, at which time they were censored at the last date they were documented to have been alive. For survival analysis, univariate Cox proportional hazard model was used to determine if amounts of fatty acids (continuous variable) were associated with the number of days of survival. Given the small sample size, fatty acids that were associated with the number of days of survival with p-value less than 0.1 were selected for optimal stratification and Cox regression analysis (categorical variable). Optimal stratification, a statistical method similar to receiver operator curve analysis, was used to solve specific threshold values within continuous variables. Optimal stratification is based on log-rank statistics that best separate patients with respect to time to an event outcome (death)^23^. This method identifies cut-points, to establish a level of fatty acids that best separate patients at risk with respect to survival. It is appropriate to determine survival-related threshold values empirically using statistical methods such as optimal stratification, where the relationship with survival for that covariate is not known (i.e. fatty acids in skeletal muscle phospholipids). In our study, we determine the cohort specific threshold amounts of phospholipid fatty acids; thresholds for these fatty acids were examined by BMI, age, tumor site and metastasis and sex. Number of days of survival were defined as days to death from the date of surgery or as number of days between date of surgery and date of data collections (for patients with censored survival time). The Kaplan–Meier method^24^ was used to establish the effect of the association between the quantity of phospholipid fatty acids on the number of days of survival. Log-rank tests were used to compare the survival curves for each variable (p < 0.05). Variables were entered into a univariate Cox proportional hazards model with 95% confidence intervals. Models were adjusted for BMI, age, tumor site and metastasis and sex. Statistical significance was reported when p-value < 0.05. Data are reported as mean ± SD. Levels of significance are p values < 0.05. All statistical analyses were performed using SPSS 20.0 (Chicago, IL, USA) for Windows.

## Results

The association of fatty acids with survival was first determined (Table 2). Univariate Cox regression analysis revealed association between overall survival and palmitoleic acid (16:1), AA, EPA, DPA (docosapentaenoic acid) and DHA with p-value < 0.1 (Table 2). Cut-points were then derived using optimal stratification for those fatty acids that were trending significant (p < 0.10). Using the cut-point value, the number of deaths and length of survival were determined for each of these fatty acids (Table 3). In the univariate analysis, median survival was in range of 450–500 days for patients having AA, EPA and DHA in muscle phospholipid below the established critical threshold compared 720–800 days for patients above (Table 3). Sixty-three percent of patients with AA, EPA and DHA depletion (below the cut-point) died within 2 years (median overall survival of 16 months). In contrast, 69% of patients with palmitoleic acid above 20.5 ng/mg in muscle phospholipids died (median survival of 16 months). Univariate Cox regression analysis for categorical variables revealed that having low levels of AA, EPA and DHA poses survival risk (HR 3.45–4.30; p ≤ 0.04) whereas palmitoleic acid below the cut-point is protective for survival (HR = 0.34; p < 0.05). BMI, age, tumor site and metastasis did not influence survival but being female was significant predictor of shorter survival (HR 2.7 (1.1–7.2), p = 0.04). The risk of death remained significant for AA [HR 3.5 (1.11–10.87), p = 0.03], EPA [HR 3.92 (1.1–14.0), p = 0.04] and DHA [HR 4.08 (1.1–14.6), p = 0.03] when adjusted for sex in the multivariate model.

**Table 2:** Univariate Cox regression analysis for each fatty acid contained in skeletal muscle phospholipid.

	Univariate Cox regression analysis (continuous variable)	
	p-value	HR (95% CI)
Palmitic acid (16:0)	0.866	1.00 (0.99–1.00)
Palmitoleic acid (16:1)	0.078	1.05 (0.99–1.11)
Stearic acid (18:0)	0.506	0.99 (0.99–1.00)
Oleic acid (18:1)	0.341	1.00 (0.99–1.01)
LA (18:2n-6)	0.175	0.99 (0.99–1.00)
GLA (18:3n-6)	0.791	1.02 (0.87–1.18)
ALA (18:3n-3)	0.739	0.96 (0.78–1.18)
DGLA (20:3n-6)	0.107	0.96 (0.92–1.00)
AA (20:4n-6)	0.089	0.99 (0.98–1.00)
EPA (20:5n-3)	0.061	0.91 (0.82–1.00)
DPA (22:5n-3)	0.090	0.96 (0.92–1.00)
DHA (22:6n-3)	0.089	0.96 (0.93–1.00)
Total MUFAs	0.197	1.00 (0.99–1.01)
Total omega-3	0.078	0.98 (0.97–1.00)
Total omega-6	0.123	0.99 (0.99–1.00)
Total SFA	0.767	1.00 (0.99–1.00)

HR are derived from univariate analysis adjusted by sex. Fatty acids with p-value < 0.1 were selected for Cox regression analysis (categorical variable) to determine threshold values of fatty acids content that best separates patients risk with respect to survival.

*ALA* alpha linolenic acid, *AA* arachidonic acid, *DGLA* Dihomo-gamma-linolenic acid, *DHA* Docosahexaenoic acid, *EPA* Eicosapentaenoic acid, *GLA* gamma linolenic acid, *LA* linoleic acid, *MUFA* Monounsaturated fatty acids, *SFA* Saturated fatty acids.

**Table 3:** Deaths, the median number of days of survival and odds ratio for fatty acids below or above level of skeletal muscle phospholipid fatty acids in cancer patients.

	Cut-point level^a^ (ng/mg)	Below cut-point			Above cut-point			Cox regression analysis (categorical variable)	
		No. of patients, N	Event, N (%)	Survival time (median)	No. of patients, N	Event, N (%)	Survival time (median)	HR (95% CI)	p-value
Palmitoleic acid (16:1)	20.5	17	5 (29)	642	16	11 (69)	493	0.34 (0.11–0.99)	0.049
AA (20:4n-6)	136.9	19	12 (63)	478	14	4 (28)	742	3.45 (1.1–10.8)	0.033
EPA (20:5n-3)	7.2	20	13 (65)	480	13	3 (23)	719	4.39 (1.2–15.5)	0.022
DHA (22:6n-3)	25.2	21	13 (62)	477	12	3 (25)	806	4.30 (1.2–15.3)	0.024

*AA* arachidonic acid, *DGLA* Dihomo-gamma-linolenic acid, *DHA* Docosahexaenoic acid, *EPA* Eicosapentaenoic acid.

^a^Cut-points were defined by optimum stratification. BMI, tumor site, metastasis and age were not significant predictors of number of days of survival.

**Figure 1:**
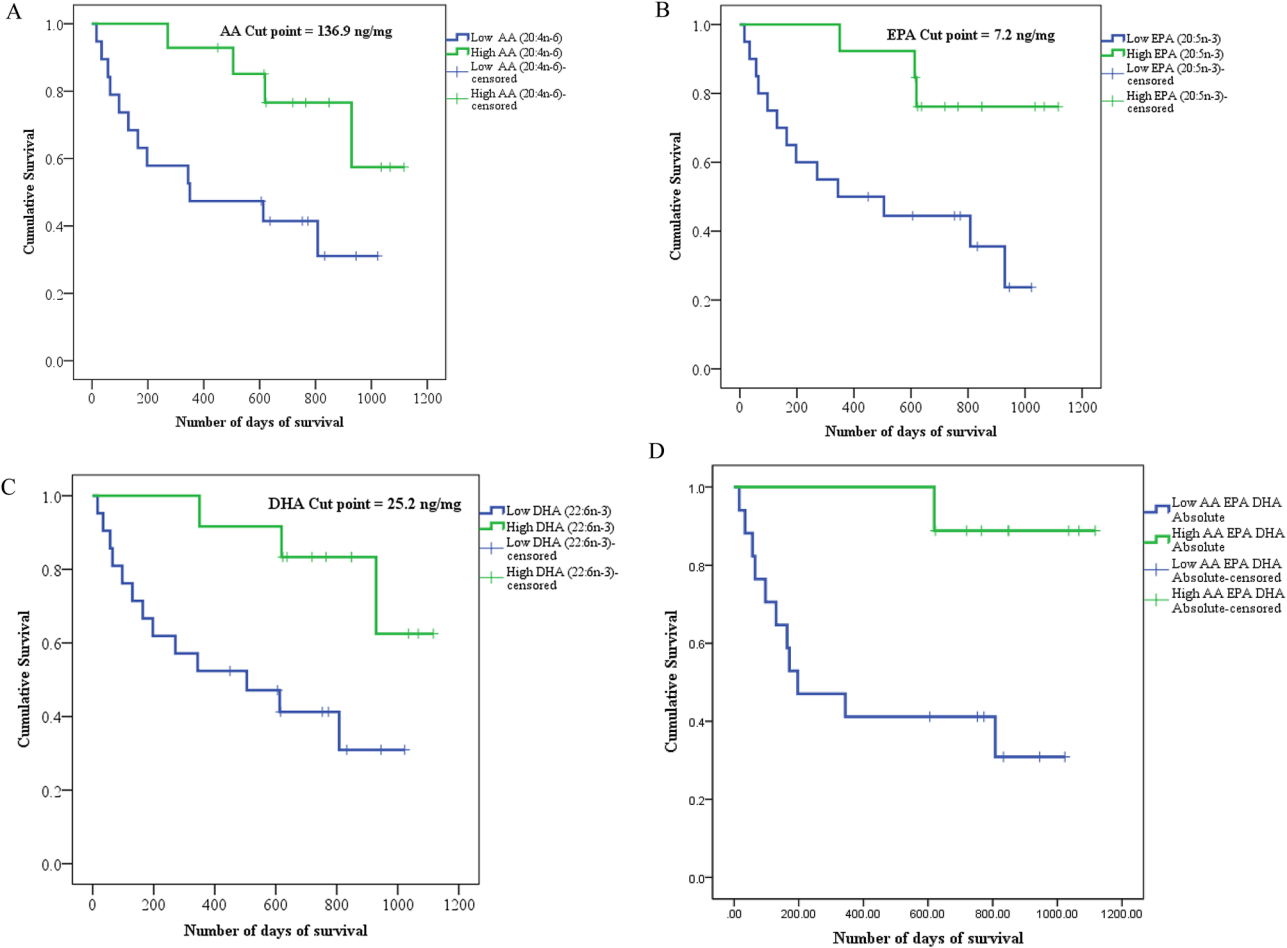
Kaplein–Meier survival curve for patients with low versus high fatty acid content in skeletal muscle phospholipids. (**A**–**C**) Represent survival distribution of surgical patients with gastrointestinal cancer based on fatty acid cut-points associated with increased mortality risk obtained by optimum stratification and adjusted for sex. (**D**) Represents survival distribution of patients with ARA, EPA and DHA above and below the cut-points. Log-rank tests were used to compare the survival curves of each variable (p < 0.05).

The survival distributions for patients below and above the cut-points for the essential fatty acids and adjusted for sex were statistically significantly different (p < 0.05; Fig. 1). The patients who had depletion of the essential fatty acids, AA, EPA and DHA simultaneously (all fatty acids below the cut-point), were compared to those with fatty acids above the cut-points to reveal that only one patient with these fatty acids above the cut-point had died at time of censorship, whereas 11 out of 17 (64%) patients with depleted levels of these fatty acids in muscle phospholipids died, ten of which died within a year of surgery.

When fatty acid data was expressed as a relative proportion, similar relationships were revealed. Having palmitoleic above, and EPA and DHA below, the respective cutpoints was significantly associated with shorter survival (Supplemental material Table 1).

## Discussion

The main goal was to determine associations between fatty acid content and composition in skeletal muscle phospholipids and survival. The most significant finding is that having a lower mean absolute amount of the long chain essential fatty acids, AA, EPA and DHA in skeletal muscle phospholipids, is associated with poor prognosis in cancer patients, with a shortened life expectancy by 200 days (5 months).

In a cohort of patients with depletion of AA, EPA and DHA below a critical level as determined by statistical cut-points, approximately 40% more patients died compared to those with fatty acids above 136.9 ng/mg, 7.2 ng/mg and 25.2 ng/mg, respectively in muscle phospholipids. Notably, only one patient died in the group of patients with AA, EPA and DHA above this critical level but 600 days post-surgery. In contrast, over half of patients died within one year of surgery in the group of patients with AA, EPA and DHA below the critical level. Two studies have previously reported an association between levels of AA, EPA and DHA of plasma phospholipid and survival. Lower content of AA, EPA and DHA were reported in plasma of cancer patients who lived < 238 days as compared to > 238 days, determined to be the median days of survival in that study population^9^. Also, longer survival of pancreatic cancer patients has been associated with higher content of EPA and DHA, but not AA, in plasma^16^. Lower proportions of AA, EPA and DHA in plasma and erythrocyte phospholipids have been reported in bladder, lung, colorectal and pancreatic cancer as compared to healthy or non-malignant populations^8,9,13,14,16^. Proportions of AA, EPA and DHA in skeletal muscle in our cohort of cancer patients were lower compared to reference range of healthy person^25,26^. Here, we demonstrate a reduction in EPA, DHA and AA in muscle and further associate this depletion with truncated survival.

Muscle loss^27,28^ and plasma phospholipid fatty acid depletion^9^ have each been separately reported to be predictors of survival in cancer. The positive association between skeletal muscle mass and concentration of EPA and DHA in plasma phospholipids has been previously reported^9^. Based on this, it was hypothesised that there would be a positive association between essential fatty acids and skeletal muscle index, however, this was not observed.

A strong association is observed between essential fatty acids in phospholipid of muscle. Membrane phospholipid fatty acids regulate the biophysical properties of proteins, provide substrates for second messengers and intracellular signals to alter gene expression. Evidence suggests that EPA and DHA in membrane phospholipids can have a protective effect against cancer through several actions including protein kinase activation, enhancing cell apoptosis and modulating inflammation^29–31^. The association between dietary/plasma AA and the risk of cancer is highly controversial^32^. AA and its metabolites play role in muscle growth^33^. AA is converted into two series eicosanoids known as prostaglandins F2alpha (PGF2α) and prostaglandin E2 (PGE2) which activate the major anabolic pathway in muscle, phosphoinositide 3-kinase (PI3K)/Akt/mammalian target of rapamycin (mTOR) signalling pathway and induce myotube hypertrophy^34,35^. Suppression of PGF2α in the muscle of experimental models has been shown to inhibit muscle recovery after disuse atrophy^36^. EPA and DHA in skeletal muscle membranes have been suggested to influence membrane properties that may influence anabolic signalling, particularly through key regulators of muscle protein synthesis^37^, and given the association with survival, these mechanisms are worthy of exploration.

Several limitations of the current study warrant mention. First, dietary records were not available for the patients, therefore, the effect of diet on skeletal muscle composition was not determined. The western diet^38,39^ and food choices cancer patients make during their cancer trajectory are foods that are good sources of AA, and its precursor linoleic acid^40,41^, therefore, depletion of AA observed is not likely to be related to intake, but rather a change in metabolism of essential fatty acids. Moreover, studies reported alterations in plasma and erythrocyte fatty acid composition in cancer patients compared to healthy controls independent of total calorie and fat intake^12,42^. Second, reliability of optimal stratification in small sample size is low^23,43^. The thresholds determined in this study should be considered approximate until confirmed in additional studies with larger sample size. While the first and only study to evaluate the PL composition in relation to survival, the sample size is too small to adjust for several well-known prognostic factors, which should be accounted for in future studies. There are several covariates that can be considered in the oncology setting, including tumor stage which is known to impact survival^44^. We did not see significant association between tumor site/metastasis and survival in our cohort. This can be due to small sample size and there is a chance of type II error. Ours is the preliminary study that provides new information that can be used to adequately power a future study to adjust for all covariates.

In conclusion, patients with AA, EPA and DHA amounts in muscle phospholipids that were below a critical level had shorter survival. The present study is a first step in establishing alterations in skeletal muscle fatty acid composition and its association with survival in cancer. This work provides rationale for conducting further studies to examine repletion of bioactive fatty acids in cancer. Further work is needed to investigate mechanisms that can explain the strong associations between essential fatty acids and survival in cancer patients observed in this study.

## Supplementary Information

Supplementary Information.

Supplementary Table 1.

## Data Availability

Authors can confirm that all relevant data are included in the article.

## Abbreviations

AA: Arachidonic acid
CT: Computed tomography
DPA: Docosapentaenoic acid
HR: Hazard ratio
PL: Phospholipid
