# Supplementary material for: Depletion of essential fatty acids in muscle is associated with shorter survival of cancer patients undergoing surgery-preliminary report
Source: Sci Rep. 2021 Nov 26;11:23006. doi: 10.1038/s41598-021-02269-0 (PMC8626431; doi:10.1038/s41598-021-02269-0)
Supplement: Supplementary file 1 — Supplementary Information. [file 41598_2021_2269_MOESM1_ESM.docx]

Appendix 1: Fatty acid composition profile – Percentage and Absolute

| **Percentage** | |  |  | **Absolute**   (ng/mg) | | |
| --- | --- | --- | --- | --- | --- | --- |
|  | Total | |  |  | Total | |
|  | Mean | Std. Deviation |  |  | Mean | Std. Deviation |
| c14:0 PL | 1.2 | 1.1 |  | c14:0 PL | 20.7 | 22.2 |
| c15:0 PL | 0.4 | 0.2 |  | c15:0 PL | 6.7 | 2.7 |
| c16:0 PL | 26.6 | 4.3 |  | c16:0 PL | 471.2 | 110.7 |
| c16:1 PL | 1.2 | 0.6 |  | c16:1 PL | 21.5 | 7.8 |
| c18:0 PL | 16.7 | 2.8 |  | c18:0 PL | 294.8 | 73.0 |
| c18:1 PL | 12.1 | 3.2 |  | c18:1 PL | 213.6 | 50.6 |
| c18:2 PL | 26.9 | 6.1 |  | c18:2 PL | 476.9 | 200.0 |
| c18:3n6 PL | 0.4 | 0.3 |  | c18:3n6 PL | 6.4 | 5.4 |
| c18:3n3 PL | 0.4 | 0.1 |  | c18:3n3 PL | 6.8 | 2.2 |
| c20:0 PL | 0.2 | 0.1 |  | c20:0 PL | 3.7 | 1.0 |
| c20:3n6 PL | 1.4 | 0.5 |  | c20:3n6 PL | 24.9 | 12.1 |
| c20:4 PL | 8.4 | 3.6 |  | c20:4 PL | 148.2 | 74.0 |
| c20:5 PL | 0.5 | 0.4 |  | c20:5 PL | 8.4 | 7.2 |
| c22:0 PL | 0.7 | 0.2 |  | c22:0 PL | 12.2 | 2.6 |
| c22:4 PL | 0.4 | 0.3 |  | c22:4 PL | 7.7 | 5.2 |
| c22:5 PL | 1.1 | 0.5 |  | c22:5 PL | 19.5 | 14.1 |
| c22:6 PL | 1.5 | 0.8 |  | c22:6 PL | 27.1 | 16.7 |
| n-6 PL | 38.1 | 8.4 |  | n-6 PL | 674.7 | 305.9 |
| n-3 PL | 3.6 | 1.6 |  | n-3 PL | 63.0 | 41.9 |
| n6/n3 PL | 10.7 | 4.4 |  |  |  |  |
